# Rat-Tail Models for Studying Hand-Arm Vibration Syndrome: A Comparison between Living and Cadaver Rat Tails

**DOI:** 10.3390/vibration7030038

**Published:** 2024

**Authors:** Christopher M. Warren, Xueyan S. Xu, Mark Jackson, Walter G. McKinney, John Z. Wu, Daniel E. Welcome, Stacey Waugh, Phillip Chapman, Erik W. Sinsel, Samantha Service, Kristine Krajnak, Ren G. Dong

**Affiliations:** 1 Physical Effects Research Branch, Health Effects Laboratory Division, National Institute for Occupational Safety and Health (NIOSH), Morgantown, WV 26505, USA; 2 Health Effects Laboratory Division, National Institute for Occupational Safety and Health (NIOSH), Morgantown, WV 26505, USA

**Keywords:** hand-arm vibration, hand-transmitted vibration, rat-tail vibration model, vibration-induced white finger, hand-arm vibration syndrome

## Abstract

Over-exposure of the hand-arm system to intense vibration and force over time may cause degeneration of the vascular, neurological, and musculoskeletal systems in the fingers. A novel animal model using rat tails has been developed to understand the health effects on human fingers exposed to vibration and force when operating powered hand tools or workpieces. The biodynamic responses, such as vibration stress, strain, and power absorption density, of the rat tails can be used to help evaluate the health effects related to vibration and force and to establish a dose-effect relationship. While the biodynamic responses of cadaver rat tails have been investigated, the objective of the current study was to determine whether the biodynamic responses of living rat tails are different from those of cadaver rat tails, and whether the biodynamic responses of both living and cadaver tails change with exposure duration. To make direct comparisons, the responses of both cadaver and living rat tails were examined on four different testing stations. The transfer function of each tail under a given contact force (2 N) was measured at each frequency in the one-third octave bands from 20 to 1000 Hz, and used to calculate the mechanical system parameters of the tails. The transfer functions were also measured at different exposure durations to determine the time dependency of the response. Differences were observed in the vibration biodynamic responses between living and cadaver tails, but the general trends were similar. The biodynamic responses of both cadaver and living rat tails varied with exposure duration.

## 1. Introduction

Workers frequently operating high-vibration tools for months or years may develop a series of vascular, neurological, and musculoskeletal disorders in their fingers and hands, collectively referred to as hand-arm vibration syndrome (HAVS) [1,2]. Cold-induced finger blanching, or vibration white finger (VWF), is the hallmark symptom of vibration-induced finger disorders. Although many studies on the disorders associated with exposure to hand-transmitted vibration have been published, the etiology of these disorders is not sufficiently understood [3,4]. The vibration exposure levels (magnitude, frequency dependence, and exposure duration) required to cause these disorders have not been clearly identified [5]. It is unclear how the contact pressure, which is caused by grip or local finger contact, affects the development of the disorders. A reliable dose-effect relationship of these vibration-induced disorders has not been established [6].

To mimic the fingers’ mechanical environment when gripping or pushing on a vibrating tool or workpiece, a new rat-tail model has been developed to study the biological responses to the combined effects of vibration and contact force [7]. Unlike previous rat-tail models, this model includes a loading device designed to apply quasi-static and vibration forces on a portion of the rat tail (typically from the C12 to 18 vertebrae) that is secured on a vibration platform fixed on a shaker or vibration tool [7,8]. Preliminary tests suggest that this new model can provide a reasonable simulation of the biodynamic responses of the human fingers in contact with a vibrating surface. Also, the vibration exposure of the rat tail can be conveniently and reliably quantified by measuring the response of the loading device. Cadaver rat tails were used in a previous test [7], but the differences in the biodynamic responses under load of the living rat tails and the cadaver rat tails have not been analyzed. In another study, the mechanical behaviors of rat tails under quasi-static loading were found to vary with the number of loading cycles and exposure duration [9]. Consequently, the biodynamic responses of the cadaver rat tails may also be time-dependent, which has not been investigated.

Rat-tail models provide a useful tool to investigate biological and/or biodynamic responses to the combined effects of vibration and contact force, mimicking the human fingers’ mechanical environment when gripping or pushing onto a vibrating tool or workpiece. Since both living rat-tail models and cadaver rat-tail models have been used to study the mechanism of VWF, it is important to understand the differences in the biodynamic responses between the living rat tails and the cadaver rat tails under vibration exposures. The hypothesis of the current study is that the biodynamic responses of living and cadaver rat tails are different but have a similar trend; furthermore, the biodynamic responses of rat tails vary with exposure duration. To test this hypothesis, this study aims to evaluate the exposure testing stations used in the experiments, to identify the differences between the biodynamic responses of cadaver and living rat tails using the new rat-tail model [7], to determine the variations in biodynamic response with exposure duration, and to assess the effects of exposure testing stations on the responses of individual tails.

## 2. Materials and Methods

### 2.1. Rat-Tail Vibration Exposure System and Modeling Method

**Figure 1.**
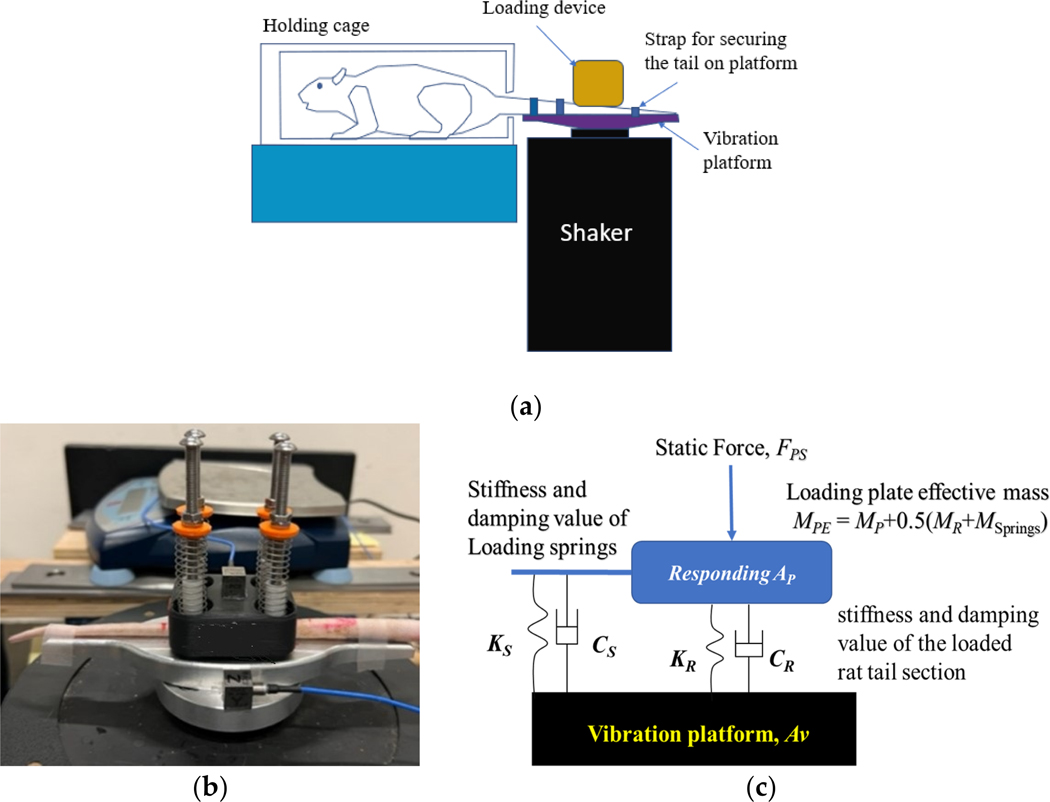
A rat-tail vibration exposure system: (**a**) general system setup; (**b**) loading device for applying static and dynamic force on the tail; (**c**) an analytical model of the exposure system.

The vibration exposure system of the new rat-tail model, together with its lumped parameter model, are illustrated in Figure 1 [7]. After 5 days of acclimation to restraint, each rat was restrained in a Broome-style restrainer and its tail was secured on a vibration platform using three elastic straps. The platform was fixed on a shaker (B&K LDS V408). The loading device applied a static force ($F_{PS}$) on the middle portion (typically C12–18 vertebrae) of the tail (Figure 1b). One accelerometer was fixed on the shaker platform ($A_{V}$, PCB-353B15) to measure the vibration input from the platform using permanent glue, and another accelerometer ($A_{P}$, PCB-356B11) was fixed on the top-center area of the plate of the loading device to measure its vibration response. The static force was applied to the tail by uniformly compressing the four loading springs installed in the loading device by adjusting the nuts on the supporting poles fixed on the vibration platform to the same height. Mineral oil was applied to the plastic guides on the poles to reduce the friction force between the plate and the guides.

Four testing stations of the vibration exposure system were built in-house and used in this study, with one rat tail on a station at a time. The four stations were simultaneously operated during the experiment. The vibration control system in this study was developed using National Instruments (NI) LabVIEW 2021. The time histories of the accelerations on the vibration platform and loading plate for each testing station were sampled at 4096 Hz. The root-mean-square (rms) value of the platform acceleration on each testing station was used as the feedback to control the vibration on the station. A MATLAB R2023b program was developed to calculate the transfer function of the loading plate using the recorded data, which is expressed as follows:

(1) $$T_{P}=\frac{A_{P}}{A_{V}}$$

As shown in the lumped parameter model in Figure 1c, the vibrating force ($F_{PD}$) acting on the loaded section of each tail depends primarily on the mass of the loading plate ($M_{P}$) and its acceleration ($A_{P}$), or $F_{PD}\approx \left(M_{PE}+0.5M_{R}+0.5M_{Springs}\right)\cdot A_{P}\approx M_{P}\cdot A_{P}$, as the plate mass (51.6 g) is much larger than the total mass of the tail section $\left(M_{R}\approx 2.32\;\text{g}\right)$ and springs $\left(M_{Springs}\approx 1.6\;\text{g}\right)$. The applied static force results in static mechanical responses such as the stress and strain of the tail tissue, and the vibration force results in the biodynamic responses (vibration stress and strain) being superposed on the static responses. This rat-tail model assumes that the biological vibration effects are associated with the combined static and biodynamic responses; accurate quantification of the biodynamic responses may help establish a reliable relationship between the vibration exposure dose and health effects [7]. The static responses can be estimated from the applied static force and the tail-platform contact area that can be directly measured, while the biodynamic responses can be estimated from the transfer function of the loading plate [7]. Hence, the transfer function can be used to represent the biodynamic responses and to examine their characteristics and influencing factors. For this reason, this study focused on the measurement and simulation of the transfer function.

The measured transfer function was simulated using the analytical model, as illustrated in Figure 1c, which can be simplified as a lumped-parameter model with a single degree of freedom [7]. The simulation used the following parameters determined from the previous study [7]: loading device stiffness ($K_{S}=53\;\text{N/m}$) and damping value ($C_{S}=2.5\;\text{Ns/m}$); loading plate mass ($M_{P}=51.6\;\text{g}$); loading spring mass ($M_{Springs}=1.6\;\text{g}$); and tail section mass ($M_{R}=2.32\;\text{g}$). The tail stiffness ($K_{R}$) and damping value ($C_{R}$) were identified from the simulation of the transfer function using a curving fitting method [7]. These two parameters represent the biodynamic properties of the rat tails. The corresponding values of undamped natural frequency ($f_{n}$) and the damping ratio (ζ) of the rat-tail exposure system were also calculated using all the model parameters [9], representing the overall dynamic characteristics of the entire exposure system. These four parameters, together with the phenomena observed in the transfer functions, were used to characterize the biodynamic responses of the tails and their time dependences.

### 2.2. Tests with the Tails of Cadaver Rats

The first set of tests used 12 tails dissected from rat cadavers of animals that were between 6 and 8 weeks of age that had been used as control animals in approved experiments for studying the health effects of inhalation exposure. The dimensions of their loaded portions were measured, and the results are listed in Table 1. The rms value of the sinusoidal excitation on each of the four testing stations used in this study was 1.0 g (9.8 m/s^2^). The static force applied on each tail was 2 N. The frequency response function of the loading plate was calculated by measuring from the measured vibration input and the response at each of the frequencies from 40 Hz to 1000 Hz in the one-third octave bands. The vibration exposure and measurement at each frequency lasted 10 s. Two trials were performed for each frequency treatment.

After the frequency response test was completed, a 4 h exposure was used to examine the effect of exposure duration on the biodynamic responses of the tails. For this purpose, two frequencies (63 and 200 Hz) were used. These were the same frequencies usually selected for biological studies using a rat-tail model [10]; one frequency did not generate resonance (63 Hz) and the other did generate resonance (200 Hz). The input and response accelerations were simultaneously measured for 5 s, starting at the following time points: 0, 3, 6, 9, 12, 15, 20, 30, 40, 50, 60, 90, 120, 150, 180, 210, and 240 min. Eight tails were used in the test at 200 Hz, with four on Test Day 1 and four on Test Day 2. Because of the availability of the tails, only four tails were used for the test at 63 Hz, which was performed on Test Day 3.

### 2.3. Tests with the Tails of Living Rats

Animals: Male (n=6) Sprague Dawley rats (Hla^®^(SD)CVF^®^, approximate body weight of 200–230 g at arrival), were obtained from Hilltop Lab Animals, Inc. (Scottdale, PA, USA). All rats were free of viral pathogens, parasites, mycoplasma, Heliobacter, and cilia-associated respiratory bacillus. Upon arrival, rats were acclimated to the AAALAC International-accredited animal facilities at NIOSH for one week. The NIOSH animal facility is specific, pathogen-free, and environmentally controlled. They were housed in ventilated micro-isolator units, and supplied with HEPA-filtered laminar-flow air (Lab Products OneCage; Seaford, DE, USA), Teklad Sanichip and Shepherd Specialty Paper’s Alpha-Dri cellulose, tap water, and an autoclaved Teklad rodent diet (Harlan Teklad; Madison, WI, USA) ad libitum. Rats were housed in pairs, and under controlled light cycle (12 h light/12 h dark) and temperature (22–25 °C) conditions. One week following acclimation to the facilities, rats were randomly assigned to applied force and vibration groups. The use of animals, as well as the housing and exposure, and all other procedures performed were reviewed and approved by the CDC Morgantown Institutional Animal Care and Use Committee, and were in compliance with the Public Health Service Policy on the Humane Care and Use of Laboratory Animals and the NIH Guide for the Care and Use of Laboratory Animals.

Exposure: After acclimation to the facilities, rats were acclimated to restraint for 5 days. Acclimation to restraint was performed by putting animals into Broome-style restrainers for gradually longer lengths of time until the total time in the restrainer was 4 h. The restrainers were large enough so that animals could move but they could not turn around or rear up onto their hind legs. Acclimation to restraint was performed by starting with 1 h of exposure in the restrainer, and then increasing the length of the exposure by 1 h/day until the rats were acclimated to 4 h of continuous restraint. After 5 days of exposure to restraint, the experiment began, and animals were exposed to applied force or control conditions. The tails of rats were gently placed on the holding platform, and the pressure platform was gently lowered onto the middle of their tail (approximately at C12–18).

Six living rats were used in the tests. Their weights and tail dimensions were similar to the cadaver tails. The input vibration acceleration was reduced from 1.0 g to 0.5 g and the exposure duration on each testing day was one hour. This design was to decrease the potential of vibration injuries to the living tails so that the tails could be repeatedly tested on different days. The other testing conditions (applying 2 N static force and testing stations) were the same as those used in the cadaver tests. The procedures for measuring the frequency responses were also the same as those in the cadaver tail tests, except that three more frequencies (20, 25, 31.5 Hz) were added to the testing matrix. Unlike the time dependency test for the cadaver tails, the time dependency of the biodynamic responses of living rat tails was examined by repeatedly measuring the frequency responses in the entire frequency range (20 to 1000 Hz) for 21 rounds, which lasted for one hour. In this way, the tail stiffness and damping values as functions of the exposure duration could be determined from the modeling of the measured transfer function spectra. After the one-hour exposure was completed on each testing day, the rats were returned to their home cages until the following day. The full experiment lasted for four days so that each tail could be exposed at each of the four testing stations to examine the effect of the testing stations on the frequency responses. The animal research protocol was reviewed and approved by the Animal Use Committee of Health Effects Research Division, NIOSH.

### 2.4. Statistical Analyses

In this study, a one-way ANOVA was performed to determine significant differences between the cadaver and living rat tails for each dependent variable. The dependent variables included the response of the loading plate at each frequency, the stiffness and damping values of the rat tails, and the fundamental natural frequency and damping ratio of the rat-tail exposure system. Using the same dependent variables, this study used a repeated-measures ANOVA to determine biodynamic response change with exposure duration. The independent variables included the testing station, testing day, and round. Round was defined as a measurement taken of the response approximately every two minutes for one hour, and was treated as a repeated measure. The statistical analysis was performed using R statistical software (The R Foundation for Statistical Computing, version 4.1.1) and SAS version 9.4 for Windows. Differences were considered significant at the p<0.05 level.

## 3. Results

### 3.1. Results of the Tests with Cadaver Tails

**Figure 2.**
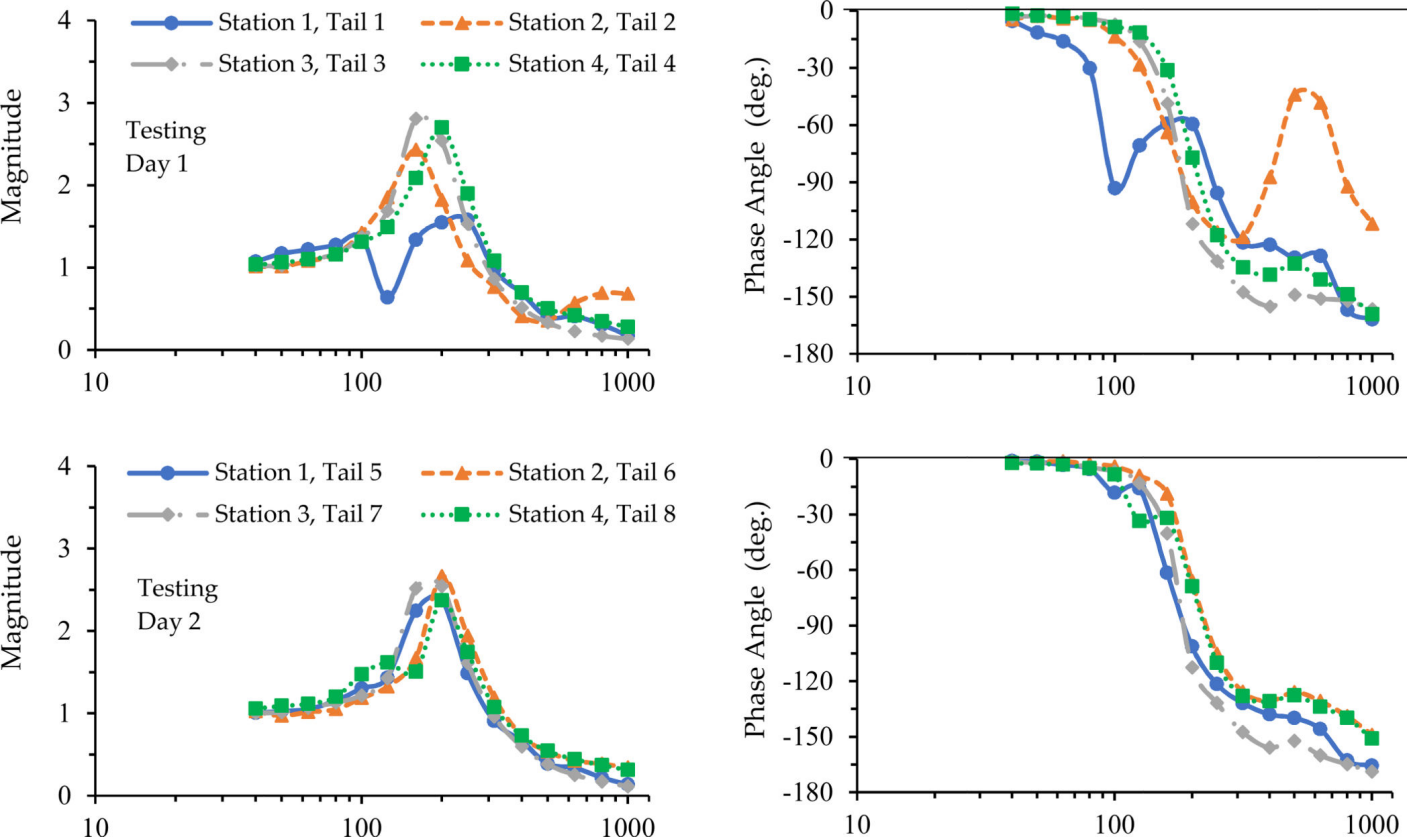

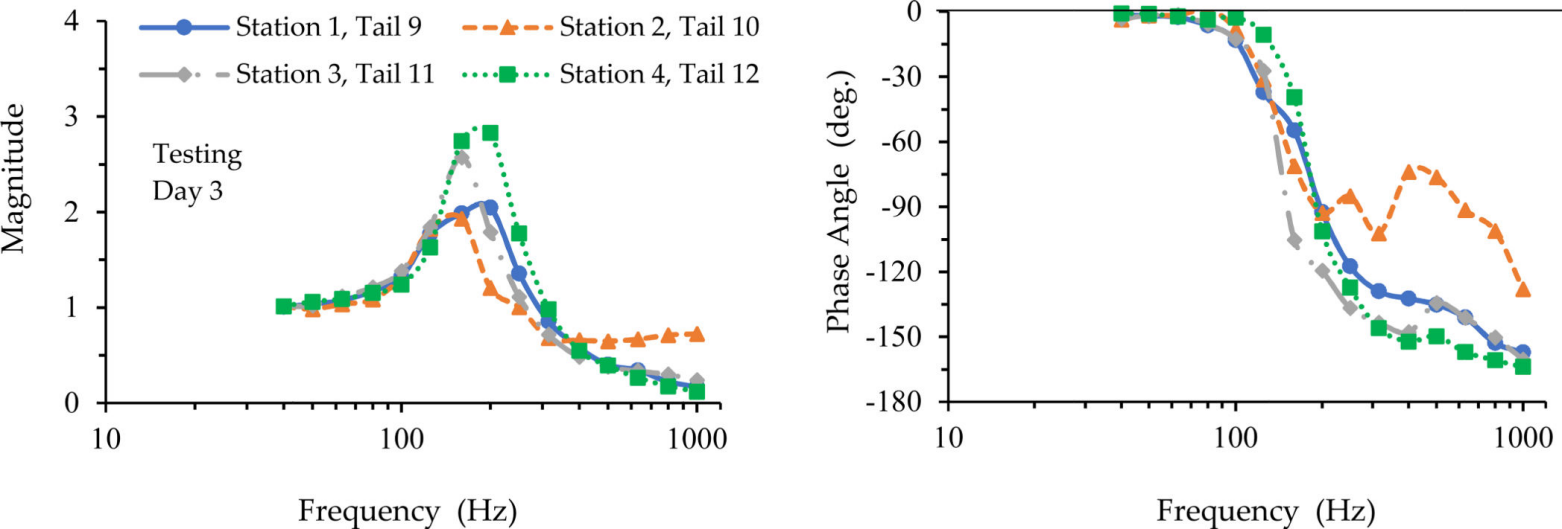
The loading plate transfer functions measured with the 12 cadaver rat tails on three testing days (with four tails on each testing day).

The plate transfer functions measured with the tails of the 12 rat cadavers are illustrated in Figure 2. As expected, the vibration responses of the loading plate generally varied by the tail, but each had a fundamental resonance generally in the range of 160 to 250 Hz, except for Tail 1. The modeling results further confirm these observations, which are listed in Table 2. The transfer functions measured with Tail 1 were obviously different from that of the other tails. It was considered an outlier and eliminated from the simulation analysis. The transfer functions of Tail 2 and Tail 10 in the high-frequency range (>300 Hz) were also quite different from those of the other tails, but their spectra in the fundamental resonant frequency range were reasonable and they were kept in the simulation analysis.

**Figure 3.**
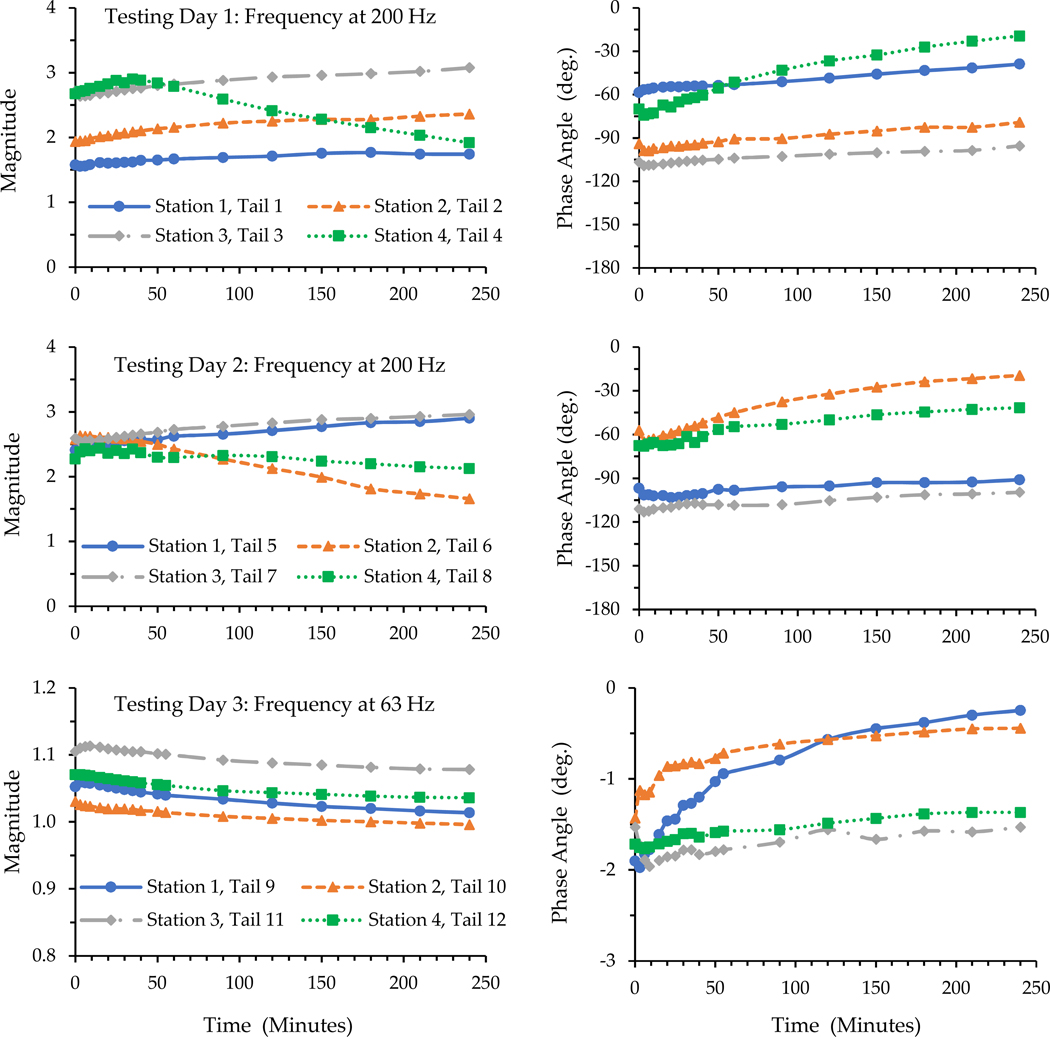
The time dependencies of the loading plate transfer functions measured with the 12 cadaver rat tails at 63 Hz and 200 Hz.

The effects of applied force and vibration exposure duration on the transfer functions at 63 and 200 Hz are illustrated in Figure 3. At the beginning, the magnitudes and phase angles measured for all the rat tails were very similar to those measured in the spectrum response tests, but they generally varied with time exposed. At 63 Hz, the changes in the magnitude and phase angle of the loading plate were very small (<1.12 and 20, respectively), and their absolute values slightly decreased with the increase in exposure duration. At 200 Hz, while the absolute values of the phase angles generally decreased with the increase in exposure duration, the changes in the magnitudes of the eight tails (Tails 1–8) did not have a consistent pattern. The response magnitude of the loading plate with Tail 4 on Station 4 generally increased during the first 35 min of exposure, but it reduced with the increase in exposure duration. While the magnitudes with Tails 1, 2, 3, 5, and 7 generally increased with the increase in exposure duration, those with Tails 6 and 8 went in an opposite direction.

### 3.2. Results of the Tests with Living Rat Tails

**Figure 4.**
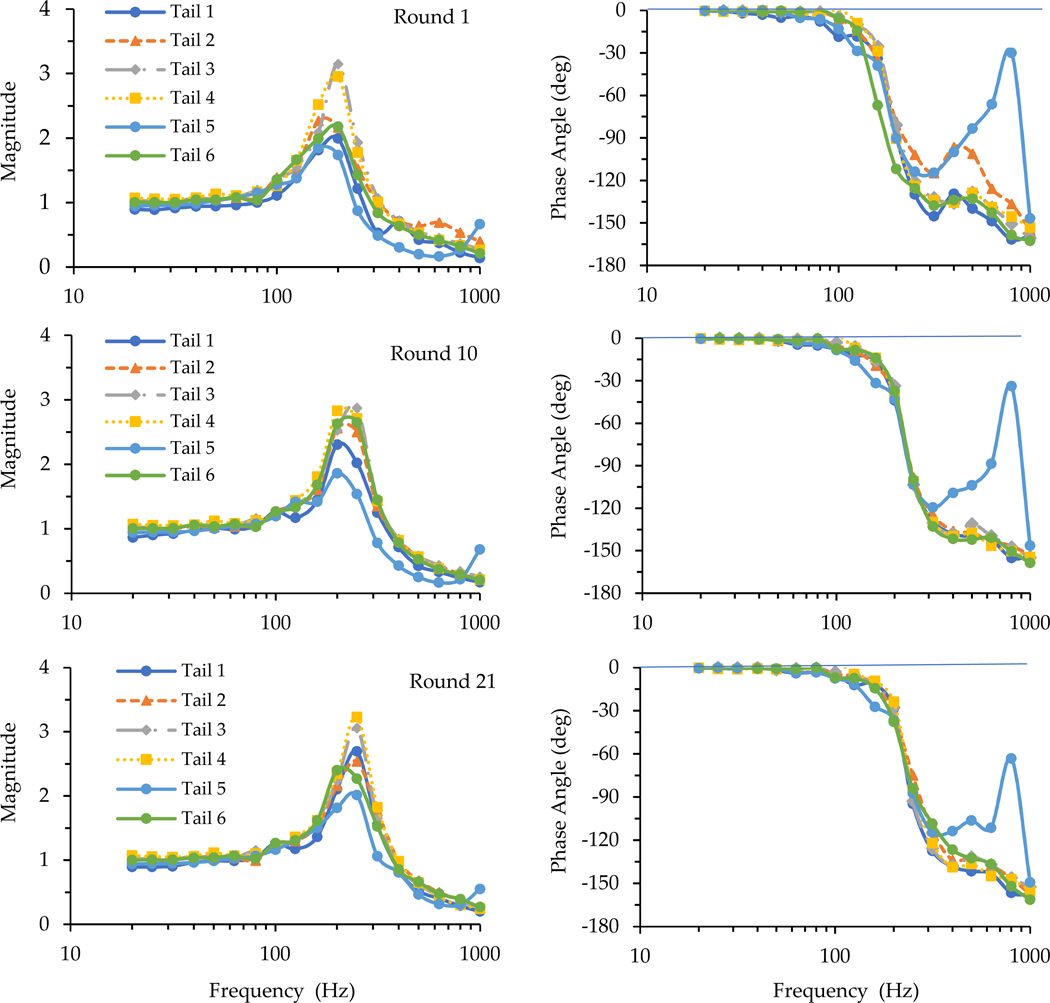
The loading plate transfer functions measured in three different (1st, 10th, and 21st) rounds of tests on the first day with the 6 living rat tails.

For demonstration, Figure 4 illustrates the loading plate transfer functions measured in the initial, middle, and end rounds of the tests on Day 1 for the tails of the six living rats. The basic characteristics of the transfer function spectra were like those illustrated in Figure 2. The resonant peak shifted to a higher frequency with the increase in testing rounds or exposure duration. The same phenomena were observed in the data measured on the remaining three testing days.

The modeling results generated from the simulation of transfer functions illustrated in Figure 4 are listed in Table 3. As they are directly comparable with those listed in Table 2, their percentage differences were also calculated and are listed in the table. Their differences were small, and none of them were statistically significant (p>0.46).

**Figure 5.**
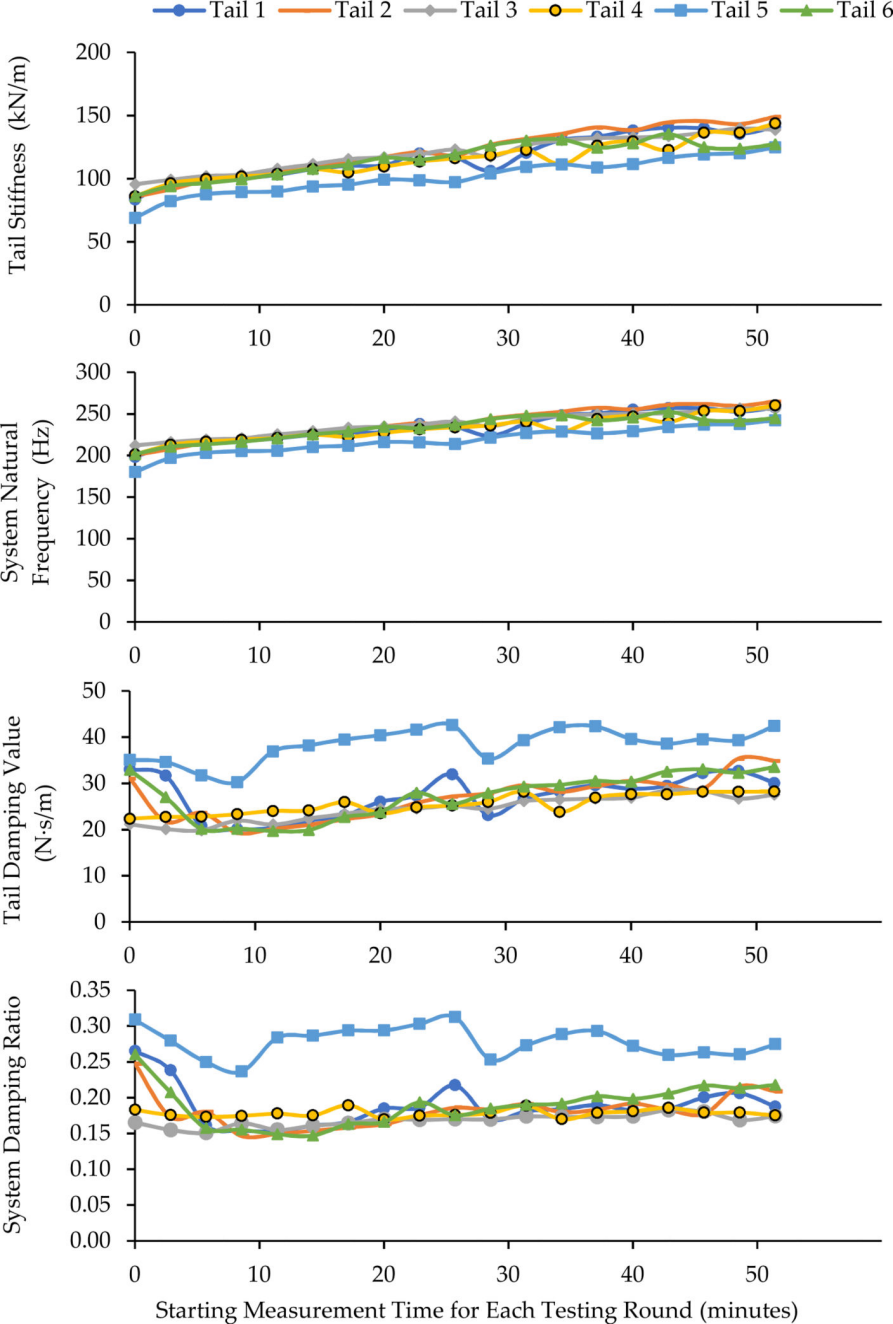
The effects of exposure duration on the stiffness and damping values of the six tails and their corresponding system’s natural frequency and damping ratio identified from the data measured on Testing Day 1.

To examine the effects of the independent variables on the transfer functions measured in the living rat tail tests, four representative parameters (the stiffness $K_{R}$ and damping value $C_{R}$ of the rat tails and their corresponding natural frequencies $f_{n}$, and damping ratio ζ of the exposure system) were identified from the simulation of the transfer function spectrum measured for each testing round. For example, the results for Testing Day 1 for each tail are illustrated in Figure 5. As also confirmed with statistical analysis, the rat tail stiffness generally increased with the number of testing rounds or exposure duration (p<0.0001). Because the undamped natural frequency of the system depends on the tail stiffness when the effective mass of the loading device is fixed, the natural frequency also increased with the increase in exposure duration (p<0.0001). However, the variations of the tail damping value and system damping ratio did not have a clear pattern, and were not significantly affected by the exposure duration (p>1.0). When the data were further analyzed by stratified testing day and testing station, some exceptions were observed. For three of the four representative parameters ($K_{R},C_{R}$, and $f_{n}$), when stratified by testing day, testing round was a significant factor (p<0.004), but the testing station and the interaction (testing round × testing station) were not significant (p>0.09). The exceptions are for damping value ($C_{R}$) on Day 3 and 4, where testing round did not show a significant effect (p>1.0). However, for damping ratio (ζ)), testing round and station, as well as their interaction, were insignificant factors (p>0.19), except on Day 1, where testing round showed a significant effect (p<0.03). Similarly, when stratified by testing station, for $K_{R},C_{R}$, and $f_{n}$, testing round was a significant factor (p<0.003), but testing day and the interaction (testing round × testing day) were not significant (p>0.07) There were two exceptions on Station 4, where testing round did not show a significant effect on $C_{R}(p>1.0$), but a significant interaction between testing round and testing day was found for $K_{R}(p<0.004$). For damping ratio (ζ), testing round and day, as well as their interaction, were insignificant factors (p>0.13), except on Station 3, where testing round showed a significant effect (p<0.006). Generally speaking, testing day, testing station, and their interactions were not significant factors.

The simulation-identified stiffness and damping values of the living rat tails for the first round of tests on each of the four days are listed in Table 4. The parameters for a tail on different stations could be very different. As displayed in Table 4, the stiffness of Tail 1 on Station 3 was about two times that on Station 4. Their damping values also varied randomly with station. Further examination of the data found that the goodness of their simulations was also quite different. The $R^{2}$-value for assessing the goodness was generally more than 0.95 for most cases (~97%), which suggests the analytical model fits the experimental data very well and the exposure system functioned as designed or expected. However, the $R^{2}$-value was less than 0.6 in some cases. In such cases, the identified tail stiffness was generally lower than others (e.g., Tail 1 on Station 4; Tail 6 on Station 4), but the tail damping value was significantly larger than those of the other tails, as shown in Table 4. In cases where the $R^{2}$-values were less than 0.60, this may indicate that the loading device might not have worked properly during such testing rounds and the measured data may not be acceptable. For this reason, the modeling results with an $R^{2}$-value less than 0.60 were eliminated from further analyses.

**Figure 6.**
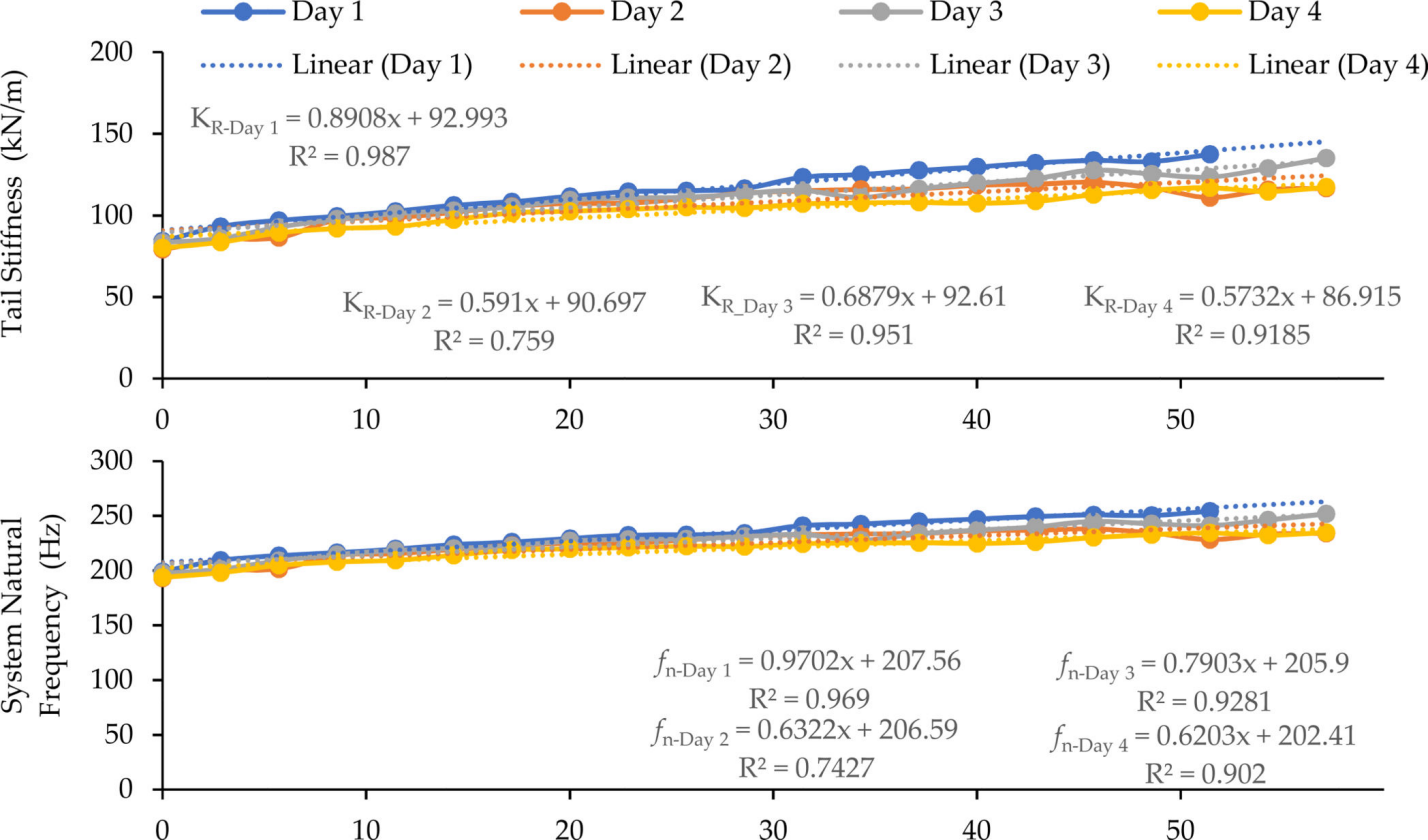

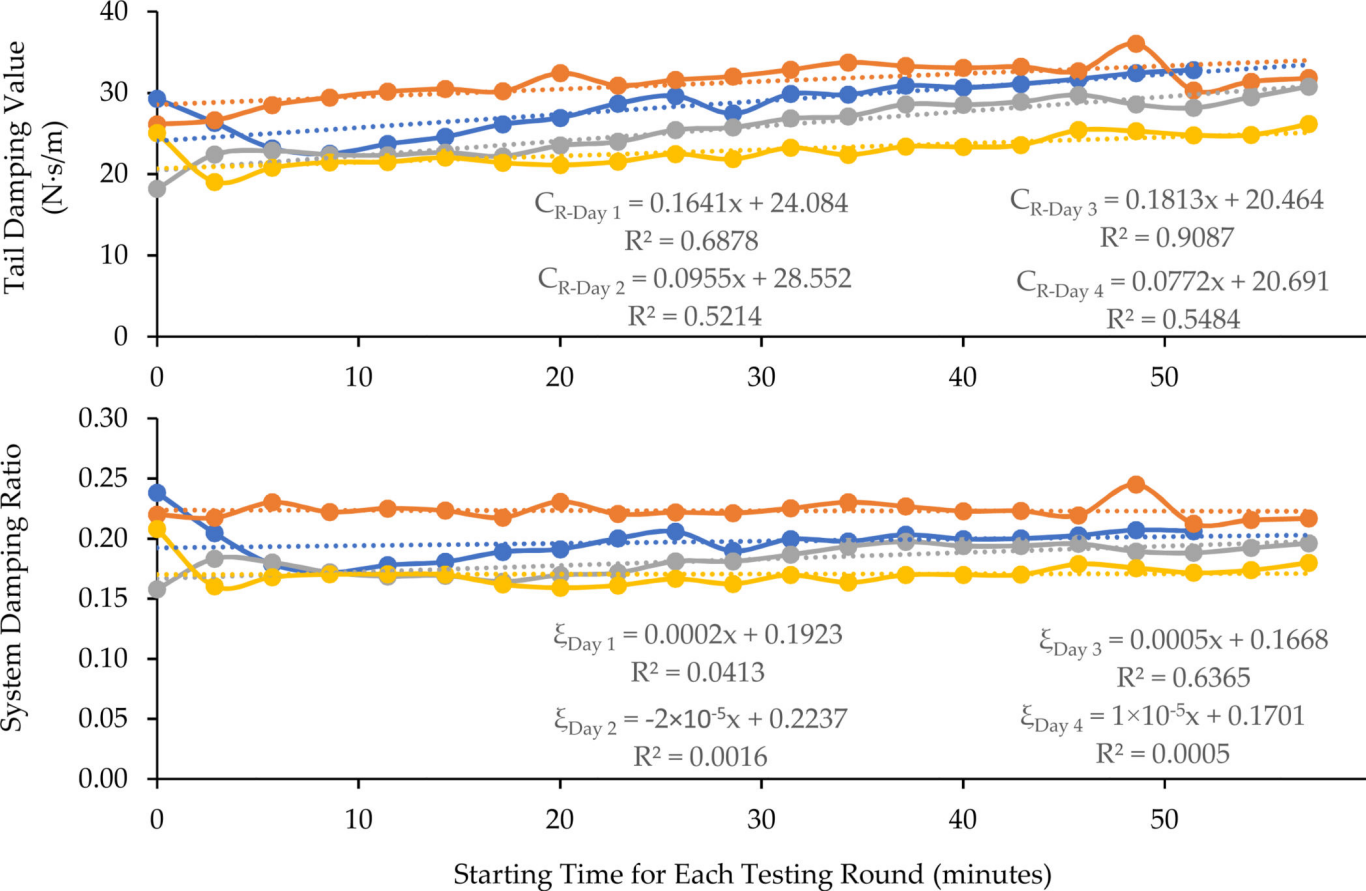
The effects of exposure duration on the daily means of the stiffness and damping values of the six tails and their corresponding system natural frequencies and damping ratios identified from the data measured during the 4 days of tests.

Figure 6 illustrates the time dependencies of the four representative parameters identified from the simulations of the transfer functions measured with the six living rat tails on each of the four testing days, together with their linear trendlines, equations, and $R^{2}$-values for assessing the goodness of the linear regression. Consistent with those observed in the data illustrated in Figure 5, the tail stiffness, damping value, and system natural frequency generally increased with the progression of testing rounds or vibration exposure duration. However, the increases in tail stiffness or natural frequency were larger than that of the tail damping value. As a result, the damping ratio did not change substantially with the increase in exposure duration because the damping ratio is proportional to the damping value, but inverse to the natural frequency [7]. It is also interesting to note that the intercepts for the starting stiffness values (93, 91, 93, and 87 kN/m) and natural frequencies (208, 207, 206, and 202 Hz) from the regression on the four different testing days were close to each other. They were significantly lower than their ending values (p<0.0001). The damping values had a large variation throughout the duration of the tests, but their starting values were significantly lower than their ending values (p<0.003), except on Day 3 and Day 4, and in the Station 4 tests from the stratified statistics (p>1.0). These observations suggest that the biodynamic properties of the tails mostly recovered to their original conditions after the rats rested for more than 20 h.

## 4. Discussion

Since both living rat-tail and cadaver rat-tail models have been used to study the mechanisms of hand-arm vibration syndrome and vibration-induced white finger, it is important to understand the functional and mechanical similarities and differences of these two types of models. This study found that the general trends and resonant features of the transfer functions of the living and cadaver rat tails’ responses to vibrations and their time-dependent characteristics were consistent, as previously reported [7]. The frequency range of the fundamental resonance under load in this study was from 160 Hz to 250 Hz, comparable to the representative scenario of fingers gripping on a vibrating tool (i.e., with a 30 N grip force [11]). This suggests that the animal models provide reasonable simulations for the finger resonant response. The observed tails’ biodynamic responses are consistent with the previous results obtained using unloaded living rat tails strapped to a platform [12]. Furthermore, the transfer functions can be closely simulated using the analytical model (Figure 1c), except in a few cases (<3%), meaning that the biodynamic responses of the tails can be quantified using the method established based on the analytical model [7]. Finally, the results showed little variation among the values measured on the four testing stations (p>0.11), confirming that the testing stations functioned as designed and can be used to conduct biological studies of tail vibration exposure.

### 4.1. The Similarities and Differences between the Biodynamic Responses of Cadaver and Living Rat Tails

As shown in Figures 2 and 4, the basic features of the transfer functions measured with the living rat tails were very similar to those of the cadaver rat tails. As shown in the data listed in Table 3, the means of the biodynamic properties identified with the transfer functions measured with the living rat tails were like those of the cadaver rat tails. As explained in the next subsection, the basic trend of the time dependency of the transfer functions measured with cadaver tails is consistent with that measured with the living rat tails. These observations confirm that the basic characteristics of the rat-tail biodynamic responses identified from the cadaver tail tests in the previous study [7] are generally applicable to living rat tails. The advantages of using cadaver rat tails in vibration exposure studies are that cadaver tails can assure more consistent vibration exposure and force measurements compared to living rat tails, and that cadaver tails are more convenient and cost-effective to use. However, this does not mean that living rat-tail models can be replaced by cadaver rat-tail models because of some fundamental differences between the two models. First, living tissues remodel in response to mechanical stimuli. Living animals can grow and adapt under repeated long-term vibration exposure. The initiation and development of HAVS are mainly due to the long-term effects of vibration exposure. Only living tails have the potential to recover or re-grow, and that is why living animal models can be used to study the mechanism of tissue degenerative development, while cadaver rat tails cannot. Secondly, the blood circulation systems of living animals help to recover the fluid distribution within the tails after mechanical loading. In comparison, the cadaver rat tails cannot recover their fluids once they are lost. Under the vibration exposure and static/dynamic force from the vibrating platform and loading plate, one acute effect was increased stiffness for both living and cadaver tails. This is due to pressing out the fluid in the tails and the compression of tail soft tissues, causing variations in the biodynamic properties of the tails in response to vibration exposure. When the exposure ends, living tails will recover, but cadaver tails do not.

### 4.2. The Time Dependencies of Rat-Tail Biodynamic Responses

The time dependencies of the biodynamic responses of rat tails can be divided into two components. The first one is termed short-term or acute time-dependency (ATD) in this study, which showed similar features with both living and cadaver tails. The applied force/pressure can gradually compress the tissues and make them ‘creep’ or deform. The vibration exposure may accelerate these processes. The results of this study demonstrate that these biomechanical processes increase the stiffness and damping values of tail tissues (Figure 5). These changes also increase the resonant frequency of the exposure system, as also shown in the figure. The increased resonant frequency shifted the entire response spectrum towards a higher frequency (Figure 4). Consequently, the phase angle below a certain frequency increased with the increase in exposure duration (Figure 3). When the excitation was below the initial resonant frequency, the response magnitude decreased with the increase in the stiffness and damping values. This explains why the magnitudes measured for Tails 9–12 at 63 Hz and those measured for Tails 6 and 8 at 200 Hz (Figure 3) generally decreased with the increase in exposure duration. When the excitation frequency was at the resonant frequency, the response magnitude could first increase with the increase in stiffness, but it decreased with further increases in stiffness or exposure duration, as observed in the responses measured with Tail 4 (Figure 3). When the excitation frequency is significantly higher than the resonant frequency, increasing the stiffness and damping values increases the response magnitude, as observed in the responses measured with Tails 1–3, 5, and 7 (Figure 3).

Unlike for cadaver tails, the changes in the tissue deformation and biodynamic properties of the living tails may gradually recover after the applied force and vibration are removed. This explains why the mean transfer functions measured on the four different days were similar, especially at the beginning of the test on each day (Figure 6). This may also be because the applied force (2 N), vibration magnitude (0.5 g), daily exposure duration (1 h) on each day, and total exposure duration (4 days) were not sufficiently large or long enough to cause substantial biological changes to the tails. If these exposure factors were increased, it is anticipated that the tail tissues may be injured, remodeled, and/or adapt to the loading environment. We also anticipate that such changes in tissue structure may result in a change in the tail’s biodynamic properties. As a result, the transfer functions measured on the first testing day may be significantly different from those measured on the last testing day. Such a change can be termed as long-term or chronic time dependency (CTD), which may be examined further with biological studies using the new rat-tail model.

### 4.3. Improvement to the Rat-Tail Vibration Exposure System

The results of this study also revealed a few technical issues with the testing stations. Besides the transfer function measured with cadaver Tail 1, the phase angles measured for cadaver Tail 2 and 10 (Figure 2) and living Tail 5 (Figure 4) in the high-frequency range (>300 Hz) differed substantially from those measured with the other tails. In these cases, the identified damping values were much larger than the other samples, as shown in Table 2 and Figure 5. The real differences between the damping values among the tails were unlikely to be so large, as the tails’ ages and dimensions were similar. Then, this suggests that the abnormally large damping value could be from the artifacts in the experiments, i.e., the friction force at the interfaces between the loading plate and the guide poles. As the damping value of the loading device $\left(C_{S}=2.5\;\text{N}\cdot \text{s/m}\right)$ was fixed in the simulation, increased damping due to friction was not accounted for in the simulation. This partially explains the high covariance in Table 2, in addition to the inter-animal differences. Such an issue is likely to become more serious with increasing applied force. The problem can be resolved using one of the following approaches, or their combination:

- Confirm that the loading devices work normally during each test by continuously monitoring the transfer function or loading plate response. A warning signal should be designed in exposure software. The rat for each testing station should not be changed so that the measured responses are directly comparable. If an abnormal or unreasonably large variation is detected, the testing station should be checked. If the loading plate is locked by friction, it can be released by adding lubricant to the frictional interfaces and/or adjusting the position/angle of the loading plate. The living tail may change its position, which could result in changes in the loading portion and the plate response. This can be corrected by adjusting the tail position and loading portion.
- Redesign the loading device by eliminating the friction interfaces. The vibration displacements in the frequency range of interest are usually small. For small displacements, a flexible support structure can be applied to replace the friction interfaces for holding the loading plate while allowing the required displacements.

Besides the above-discussed issues, the large variation in the experimental and modeling data may also partially result from inconsistencies among the four testing stations. A major improvement can be made by developing a more accurate method for applying the static loading force. Besides improving these testing stations and their relation issues, we suggest that the transfer function is continuously measured via the input and response accelerations during the entire experiment for a biological study. The measured data can be used to accurately quantify the vibration exposure dose. Tracking the biodynamic changes may also help to understand the biological effects.

## 5. Conclusions

Both living and cadaver rat-tail models provide useful tools to investigate and understand the health effects on human fingers exposed to vibration and force. The results of this study confirm that the trends in biodynamic responses and their variations with force and vibration exposure duration for living and cadaver rat tails are consistent. Tail stiffness and damping values generally increase with exposure duration; consequently, the fundamental natural frequency of the exposure system increases with exposure duration. The results of this study also demonstrate that living rat tails can recover their biodynamic properties after sufficient rest. Based on the current findings and observations, we have recommended some tactics for improving the design of the rat-tail exposure system and appropriate operation methods.

## Figures and Tables

**Table 1.** The lengths and diameters of the cadaver rat tails underneath the loading plate, or in the loaded portion $\left(L_{t}=53\;\text{mm}\right)$.

Tail ID	Proximal Diameter (mm)	Distal Diameter (mm)	Exposed Tail Segment Length (mm)	Mean Diameter $\left(d_{t}\right)$ (mm)	Tail Mass of Exposed Segment Portion $\left(L_{t}=53\;\text{mm}\right)$, $M_{R}(g)$
1	6.1	4.28	40.78	5.19	2.37
2	7.38	4.66	41.55	6.02	2.44
3	6.58	4.89	40.35	5.74	2.75
4	7.78	3.99	39.37	5.89	2.52
5	7.52	4.25	41.24	5.89	2.47
6	6.98	4.44	40.15	5.71	2.50
7	7.15	4.59	42.11	5.87	2.51
8 *					
9	6.98	4.12	43.15	5.55	2.44
10	7.51	4.5	42.22	6.01	2.38
11	7.57	3.99	43.17	5.78	2.65
12	7.24	4.36	42.11	5.8	2.50
Mean	7.16	4.37	41.47	5.77	2.50

* Dimensions for this tail were not collected.

**Table 2.** The stiffness and damping values of the rat tails and the natural frequency and damping ratio of the exposure system identified from the modeling of the plate transfer functions measured with the cadaver tails.

Tail ID	Tail Stiffness, $K_{R}$ (N/m)	Tail Damping Value, $C_{R}$ (N·s/m)	Natural Frequency, $f_{n}$ (Hz)	Damping Ratio, ζ
Tail 1 *				
Tail 2	65,103	30	175	0.27
Tail 3	72,465	18	185	0.16
Tail 4	98,133	25	215	0.19
Tail 5	86,127	28	202	0.23
Tail 6	103,644	26	221	0.19
Tail 7	83,074	24	198	0.19
Tail 8	101,416	32	219	0.23
Tail 9	82,167	33	197	0.27
Tail 10	53,040	38	158	0.38
Tail 11	64,205	23	174	0.21
Tail 12	78,008	18	192	0.16
Mean	80,671	27	194	0.23
STD	16,286	6	20	0.06
CV	0.20	0.23	0.10	0.28

* Identified as outlier and eliminated from the simulation analysis.

**Table 3.** The stiffness and damping values of the rat tails and the natural frequency and damping ratio of the exposure system identified from the simulation of the plate transfer functions measured in the first round of tests with the living rat tails.

Tail ID	Tail Stiffness, $K_{R}$ (N/m)	Tail Damping Value, $C_{R}$ (N·s/m)	Natural Frequency, $f_{n}$ (Hz)	Damping Ratio, ζ
Tail 1	83,454	33	198	0.27
Tail 2	85,586	31	201	0.25
Tail 3	95,494	21	212	0.17
Tail 4	85,912	22	201	0.18
Tail 5	68,911	35	180	0.31
Tail 6	86,176	33	202	0.26
Mean	84,256	29	199	0.24
STD	8609	6	10	0.05
CV	0.10	0.20	0.05	0.23
Difference = (Living mean – Cadaver mean)/Living mean	4.3% (Insignificant: p=0.63)	8.1% (Insignificant: p=0.46)	2.4% (Insignificant: p=0.59)	6.1% (Insignificant: p=0.64)

**Table 4.** The stiffness and damping values of the rat tails identified from the simulation of the plate transfer functions measured in the first round of tests with the living rat tails on the four testing days.

Tail Stiffness, $K_{R}$ (N/m)							
IDs	Tail 1	Tail 2	Tail 3	Tail 4	Tail 5	Tail 6	Mean
Station 1	83,454	68,971	72,340	74,470	68,911	69,305	72,908
Station 2	68,353	85,586	78,846	71,372	83,918	86,176	79,042
Station 3	107,189	82,954	95,494	93,590	83,805	80,784	90,636
Station 4	52,827 *	71,261	73,798	85,912	89,719	38,831 *	68,725
Tail Damping Value, $C_{R}$ (N·s/m)							
Station 1	33	15	18	29	35	21	25
Station 2	50	31	22	19	30	33	31
Station 3	16	18	21	25	36	25	24
Station 4	65 *	20	29	22	42	90 *	45

* Cases with poor goodness of fit ($R^{2}$-value < 0.6).

## Data Availability

The experimental data are available upon request.
